# In an Absolute State: Elevated Use of Absolutist Words Is a Marker Specific to Anxiety, Depression, and Suicidal Ideation

**DOI:** 10.1177/2167702617747074

**Published:** 2018-01-05

**Authors:** Mohammed Al-Mosaiwi, Tom Johnstone

**Affiliations:** Department of Psychology, School of Psychology and Clinical Languages, University of Reading

**Keywords:** affective disorders, depression, text analysis, cognitive style, anxiety, open data, open materials

## Abstract

Absolutist thinking is considered a cognitive distortion by most cognitive therapies for anxiety and depression. Yet, there is little empirical evidence of its prevalence or specificity. Across three studies, we conducted a text analysis of 63 Internet forums (over 6,400 members) using the Linguistic Inquiry and Word Count software to examine absolutism at the linguistic level. We predicted and found that anxiety, depression, and suicidal ideation forums contained more absolutist words than control forums (*d*s > 3.14). Suicidal ideation forums also contained more absolutist words than anxiety and depression forums (*d*s > 1.71). We show that these differences are more reflective of absolutist thinking than psychological distress. It is interesting that absolutist words tracked the severity of affective disorder forums more faithfully than negative emotion words. Finally, we found elevated levels of absolutist words in depression recovery forums. This suggests that absolutist thinking may be a vulnerability factor.

Absolutist thinking underlies many of the cognitive distortions (Beck, 1979; Burns, 1989) and irrational beliefs (A. Ellis & Harper, 1975) that are purported to mediate the core affective disorders. Words, phrases, and ideas that denote totality, either of magnitude or probability, are often referred to as “absolute.” Absolutist thoughts are independent of context and unqualified by nuance. In this observational study, we aimed to measure absolutist thinking in a specific and ecologically valid manner. We then compared its relative association between a variety of affective and nonaffective groups.

Absolutist thinking has strong empirical links to three distinct mental health groups: suicidal ideation, borderline personality disorder (BPD), and eating disorder (ED). Regarding suicidal ideation, structured response formats have shown more extreme value judgments by suicidal patients than controls (e.g., Neuringer, 1961, 1964). Thematic analysis by independent raters also deemed the stories and poetry of suicidal individuals as highly “polarized” (Litinsky & Haslam, 1998; Wedding, 2000). In addition, dichotomous thinking, cognitive rigidity, and problem-solving deficits have been repeatedly found to co-occur in suicidal individuals (for review, see T. E. Ellis & Rutherford, 2008). This is supported by a series of empirical studies from Pollock and Williams (1998, 2001, 2004; J. M. G. Williams & Pollock, 2008).

BPD patients also make more extreme responses on structured response formats than controls (e.g., Moritz et al., 2011; Napolitano & McKay, 2007; Sieswerda, Barnow, Verheul, & Arntz, 2013; Veen & Arntz, 2000). Some scholars have used “spontaneous reactions” or short interviews to identify extreme or dichotomous thinking styles (e.g., Arntz & ten Haaf, 2012; Arntz & Veen, 2001).

With respect to ED, the Dichotomous Thinking in Eating Disorders Scale (Byrne, Allen, Dove, Watt, & Nathan, 2008) is widely used in ED studies (e.g., Antoniou, Bongers, & Jansen, 2017; Palascha, van Kleef, & van Trijp, 2015). Although obesity and anorexia are often studied separately, they both link to absolutist thinking. For obesity, several reviews have found that avoiding absolutist dichotomous thinking improves weight loss maintenance (e.g., Ohsiek & Williams, 2011). Absolutism often takes the form of perfectionism in anorexia, as identified through clinical observations (e.g., Fairburn, Cooper, & Shafran, 2003; Garner, Garfinkel, & Bemis, 1982), structured response formats (e.g., Feixas i Viaplana, Montebruno, Dada, Castillo, & Compañ, 2010; Zotter & Crowther, 1991), and interviews (e.g., Johnson & Holloway, 1988).

Despite the inclusion of absolutist thinking into many cognitive therapy models for anxiety and depression (Beck, 1979; Burns, 1989; C. Williams & Garland, 2002), this association remains mostly neglected in the empirical literature (A. Ellis, 1987). In a notable exception, Teasdale et al. (2001) found that an “absolutist, dichotomous thinking style” predicted future depressive relapse, over and above the content of responses. This was evidenced by both positive and negative “extreme responses” on Likert-type scales.

Attempts to investigate absolutist thinking have mostly employed some type of structured response format. Ertel (1985) was the first to use quantitative text analysis to measure dogmatism with the manual Dogmatism Text Analysis Tool. More recently, with the advent of automated text analysis, Cohen (2012) measured “cognitive rigidity” in the “spontaneous autobiographical narratives” of undergraduate students and found correlations with negative emotionality. Unlike structured response formats, these natural language text analysis studies have more ecological validity.

With the growth of social media, Internet forums are increasingly being used as a source of naturalistic writing for research in depression and other affective disorders (e.g., Fekete, 2002; Griffiths, Calear, & Banfield, 2009; Houston, Cooper, & Ford, 2014). It is believed that insights into the cognitive processes associated with particular affective disorders can be gleaned from how people with those disorders write about their experiences. In three connected studies, we investigated the frequency of absolutist words contained in different affective and nonaffective Internet forum groups (Table 1; for more details, see Table S1 in the Supplemental Material available online). In the first study we compared anxiety, depression, and suicidal ideation (test) groups with general, asthma, diabetes, and cancer (control) groups. We had two specific hypotheses:

**Table 1.** Characteristics of Test and Control Internet Forums

Study	Condition	Group	Forums^b^	Members^c^
Study 1	Control	General^a^	7	917
		Asthma	4	418
		Diabetes	4	587
		Cancer	4	451
	Test	Anxiety	6	597
		Depression	6	529
		Suicidal Ideation	4	368
Study 2	Control	PTSD	6	534
		Schizophrenia	6	591
	Test	BPD	4	326
		ED	5	547
Study 3		Recovery	7	558

Note: PTSD = posttraumatic stress disorder; BPD = borderline personality disorder; ED = eating disorder.

a General forums = Mumsnet (Women), Ladies Lounge (Women), Gentlemen’s Club (Men), Ask Men (Men), Pensioners Forum (Elderly), Student Room (Young), Work Problems. ^b^Number of Internet forums in each group. ^c^Number of members who contributed to that group’s corpus.

*Hypothesis 1* (*H*_1_): The percentage of absolutist words in anxiety, depression, and suicidal ideation test forum groups will be significantly greater than in Study 1 control forum groups.
*Hypothesis 2* (*H*_2_): The percentage of absolutist words in the suicidal ideation forum group will be significantly greater than in both anxiety and depression forum groups.

Our second hypothesis is partly based on the strong association between suicidal ideation and absolutist thinking (for review, see Arffa, 1983). But also, as suicidal ideation is the more severe mental health concern, it could be hypothesized that absolutist thinking will be correspondingly more extreme.

In Study 2, our aim was to show that absolutist words reflect absolutist thinking, rather than psychological distress. We attempted to control for psychological distress by comparing groups believed to have similar levels of negative emotions but different levels of absolutist thinking (Table 1 and Table S1). We compared mental health groups strongly associated with absolutist thinking (BPD and ED, cited above) with mental health groups less associated with absolutist thinking (posttraumatic stress disorder [PTSD] and schizophrenia). Although we recognize that PTSD and schizophrenia may also have some links to absolutist thinking, the literature suggests these links are likely to be much weaker than those of BPD and ED. Relatively few researchers have examined absolutist thinking in PTSD and schizophrenia, and these have often been limited or produced mixed results (e.g., Colbert, Peters, & Garety, 2010; Joseph & Gray, 2011). Conversely, there is a widespread consensus, based on a multitude of studies, that BPD and ED are firmly linked to absolutist thinking (e.g., Alberts, Thewissen, & Raes, 2012; Napolitano & McKay, 2007; Veen & Arntz, 2000). We also measured the frequency of negative emotion terms to further support the assumption that the four mental health groups had comparable levels of negative emotions.

*Hypothesis 3* (*H*_3_): The percentage of absolutist words in BPD and ED test forum groups will be significantly greater than in PTSD and schizophrenia control forum groups.

In Study 3, we aimed to determine the extent to which absolutist thinking could be a cognitive vulnerability factor for depression and suicidal ideation. In a subset of depression and suicidal ideation forums, there are “recovery” subforums (Table 1 and Table S1). These subforums are visited by members who feel they are currently out of depression. They often write very positive posts about their progress and words of encouragement to other members. Theoretically, a cognitive vulnerability factor should not only be present during an episode of depression but also persist during recovery. Therefore,

*Hypothesis 4* (*H*_4_): The percentage of absolutist words in the recovery forum group will be significantly greater than in Study 1 control forum groups.

Previous text analysis research has examined many different dictionary “dimensions.” When analyzing written samples from anxious, depressed, or suicidal individuals, an increased use of personal pronouns and negative emotion words has commonly been found (Bucci & Freedman, 1981; Fekete, 2002; Lorenz & Cobb, 1952; Rude, Gortner, & Pennebaker, 2004; Stirman & Pennebaker, 2001; Weintraub, 1981). In particular, pronouns have been identified as having a stronger relationship with affective disorder than negative emotions (Pennebaker & Chung, 2013). Like pronouns, absolutist words are functional; they help determine our style of writing, not its contents. Moreover, functional words are ordinarily outside of conscious control (Pennebaker & Chung, 2013); therefore, they can serve as implicit markers. We believe a shift in focus to how we think rather than what we think can provide greater insight into possible cognitive mechanisms underlying affective disorders.

From the outset, we identified and validated a single dictionary of interest, as this study was motivated by specific a priori hypotheses. This is in contrast to previous text analysis studies that have used a subset of already constructed dictionaries or identified features of interest based on the data itself (e.g., using an iterative process with cross-validation and feature reduction; Mladenić, 2005). The large data set in this study, from 12 different groups, representing 63 different Internet forums and more than 6,400 members, afforded a degree of ecological validity not achievable in experimental studies. However, as with many observational studies, these benefits come with inherent costs. We had limited information about the members posting in the forums, and for the most part, their true identities and motivations were unknowable. Recognizing this limitation, we hope that follow-up studies, using alternative experimental designs, will extend the findings presented here.

## Method

### Forum selection

We used English-language Internet forums as a source of naturalistic writing for our test and control categories. For all three studies, representative websites were located through a Google search (search words: e.g., “suicide forums,” “asthma forums”). Forums were selected for inclusion into the study on the basis of Google rank (Table 1 and Table S1), were popular (thus yielding sufficient data for analysis), and were actively moderated with clearly written moderation policies. Each group in the test and control categories was composed of between four to seven separate forums, as determined by forum availability. For Study 1, control groups were carefully selected to provide the broadest level of control. The “general” group provides a gender control with two forums for female members (Mumsnet and Ladies Lounge) and two for male members (Askmen and Gentlemen’s Club). The general group also controls for age, with a designated forum for young members (Student Room) and older members (Pensioners Forum). The asthma and diabetes groups control for chronic physical illness, and the cancer group controls for severe physical and psychological distress. Study 3 recovery forums were located within Study 1 depression and suicidal ideation test forums.

### Data collection

Forum members can either introduce a new topic (“first posts”) or contribute to an ongoing discussion (“replies”). In the interest of simplicity and interpretability, only first posts were collected. Posts were copied and pasted into a text document ready for subsequent text analysis. Where an individual member contributes multiple posts, these were combined into a single text document. All text files used in this study are hosted on Figshare (doi:10.6084/m9.figshare.4743715). If a forum was further divided into subforums, only the single most appropriate subforum was used (Table S1). For each test and control forum, we aimed to collect 30,000 words. Seven out of the 63 forums were not large enough to provide a 30,000-word corpus but were nevertheless retained in the study as they surpassed 10,000 words. Posts were only collected if they met our selection criteria: (a) contain a minimum of 100 words, (b) be authored by a representative member of that online community (i.e., not written on behalf of someone else/news article etc.), and (c) be written in continuous prose (i.e., not lists, poems). Posts from all test and control forums which met the selection criteria were collected sequentially as presented by the respective forum website (usually by date order). Posts were collected between April and May 2015 and December and January 2016. All data in this study was collected from the public domain; therefore, although ethical consideration is still important, informed consent is not required. This complies with the University of Reading research ethics guidelines and the ethical guidance for internet-mediated research set out by The British Psychological Society (British Psychological Association, 2013). The aggregate data used in this study are hosted on Figshare (doi:10.6084/m9.figshare.4743547.v1).

### Word count text analysis

Word counting text analysis was conducted using validated dictionaries that characterize a particular linguistic dimension (i.e., negative words, auxiliary verbs, family related words). For this study, we validated an absolutist and a nonabsolutist words dictionary using independent expert judges.

Absolutist and nonabsolutist words indicate magnitudes or probabilities; absolute words do so without nuance (i.e., always, totally, entire), whereas nonabsolute words indicate some degree of nuance (i.e., rather, somewhat, likely). Both dictionaries are composed of functional words devoid of valence, mostly adverbial intensifiers or modal verbs. A subclass of nonabsolutist words, which we have termed “extreme words,” indicate extreme (but not absolute) magnitudes or probabilities (i.e., “very”). Although the terms *extreme* and *absolute* have previously been used interchangeably (e.g., Teasdale et al., 2001), we treat them here as qualitatively distinct.

To construct these dictionaries, we initially brainstormed more than 300 absolutist words and 200 nonabsolutist words (including extreme words). Testing on pilot data (control and test groups) revealed that many of the words on these original lists were too obscure to register with sufficient frequency for analysis. Consequently, the original dictionaries were reduced to the most prevalent 22 absolutist words and 43 nonabsolutist words (including 21 extreme words). Although this was based on a mostly arbitrary cutoff, it was intended that the lists be large enough to produce representative dictionary percentages, but small enough to facilitate independent validation by experts. The 22 absolutist words and 43 nonabsolutist words were combined into a single list of 65 words. Five independent expert judges were asked to categorize them as absolute, nonabsolute, and/or extreme. Two of the judges are clinical psychologists from the University of Reading Charlie Waller Institute and three are linguists from the University of Reading School of Clinical Language Sciences. Judges were permitted to place words into more than one category (i.e., extreme and absolute). The agreement between our original categorization of the words (absolutist/nonabsolutist) and that of the judges ranged between 83% and 94%, whereas the interjudge agreement was 96%. Words were considered absolute, extreme, or nonabsolute on the basis of a majority decision by the judges. Three words, *anything, need*, and *needed*, were moved from the absolutist dictionary to the nonabsolutist dictionary as they were not categorized as absolute by the majority of judges. All the words on our nonabsolutist dictionary were judged nonabsolute. Judges showed almost no agreement on extreme words, this category was consequently removed from the analysis (collapsed into the nonabsolutist category).

The resulting 19-word absolutist dictionary is shown in Table S2 in the Supplemental Material. Both dictionaries were used in the text analysis of test and control groups. We also ran dictionaries contained within the Linguistic Inquiry and Word Count program (LIWC; Pennebaker, Booth, Boyd, & Francis, 2015). This program provides 73 validated dictionaries covering a wide range of “dimensions” (i.e., questioning words, affective processes, auxiliary verbs). All dictionaries, other than the absolutist dictionary, were run purely for the benefit of comparison.

The LIWC text analysis software was used to test our absolutist and nonabsolutist dictionaries as well as the LIWC dictionaries. It calculates the prevalence of a given dictionary as a percentage of the total number of words analyzed. Throughout, we have referred to this percentage measure of a dictionary’s prevalence as its “index.” In each forum, we calculated an index for 75 dictionaries (1 absolute, 1 nonabsolute, and 73 LIWC).

For the absolutist index we have endeavored to account for false positives. There are three principal types of false positives: a negation before the absolutist word (i.e., “not completely”), a qualifier before the absolutist word (i.e., “almost completely”), and a salutation (i.e., “hello everyone”). These would ordinarily register on our absolutist index and distort our measure of absolutism. Fortunately, the LIWC (2015 version) can also count phrases, so we ran a second version of our absolutist dictionary composed of the most common false positives (as described). The absolutist false positive index was subtracted from the absolutist index to provide a better estimate of absolutism. We nevertheless rely on the assumption that any remaining false positives are equally distributed between groups.

## Results

### Study 1

#### Data analysis

The control and test category forums were subdivided into groups as shown in Table 1. To analyze the data, a multilevel mixed-effects modeling approach was adopted (the SPSS syntax script can be found in the Supplemental Material). This is the recommended analysis method for this type of data structure (Baayen, Davidson, & Bates, 2008). Members were nested within forums, and forums were nested within groups (i.e., depression). Mixed-effects models consider both fixed and random effects and can be used to assess the influence of the fixed effects on the dependent variables after accounting for some outside random effects. Residuals were weighted by the word count of each text file and all the analysis was conducted using IBM SPSS software (version 21). To correct for positive skew in the data, we used a log10(x + 1) transformation, adding 1 to deal with 0 values (cf. Yamamura, 1999). We report raw values for descriptive statistics to facilitate a more intuitive understanding. The bootstrap procedure was also used to produce better estimates of *p* values and confidence intervals (CIs). This method is often recommended because it does not assume normally distributed data (Cumming, 2014). Bootstrapped CIs (95%; bias-corrected and accelerated) were computed through 1,000 random resamples (with replacement) using the stratified sampling method, with forums as the strata variable.

#### Control group

There was no significant omnibus effect among the control groups as determined by a multilevel mixed effects model, *F*(7, 11) = 0.754, *p* = .635 (Table 1 and Table S1). Consequently, they were combined into a single “control group.” It is important that this suggests that the absolutist index is largely independent of content, as it demonstrates remarkably little variance across a wide range of very different discussion topics.

#### Multilevel mixed-effects model for the absolutist index

**Fig. 1.**
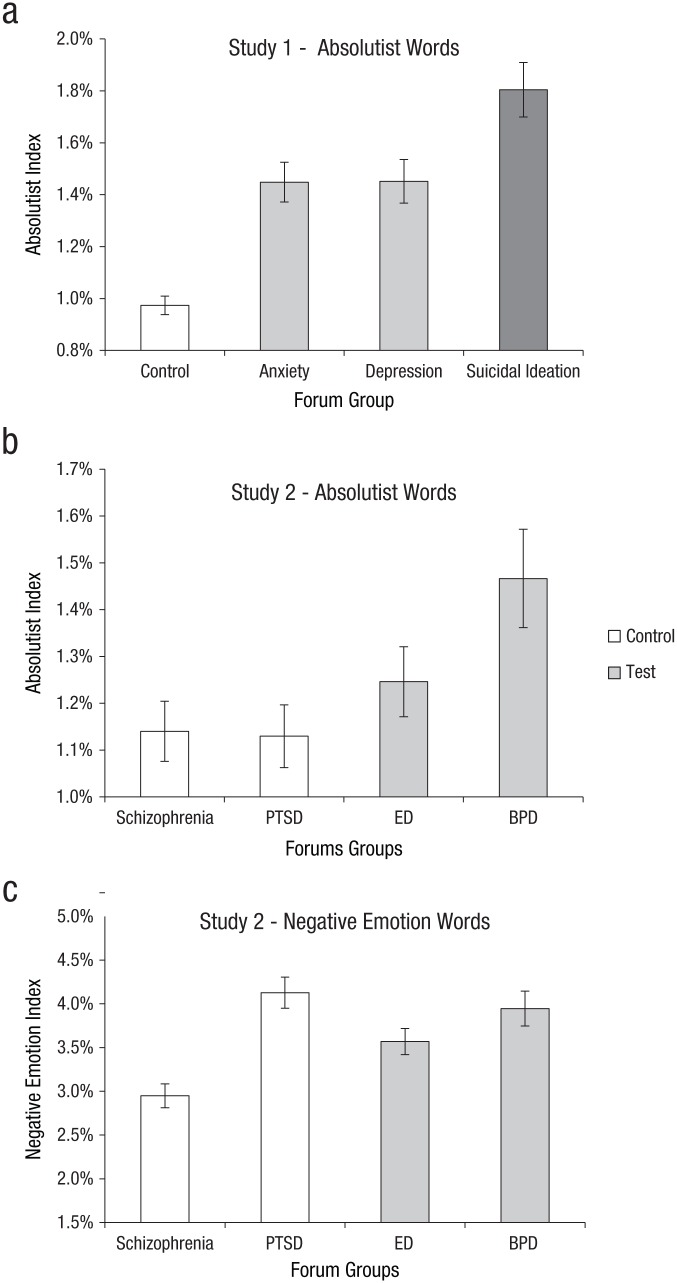
Mean percentage of (a) absolutist words in Study 1 groups, (b) absolutist words in Study 2 groups, and (c) negative emotion words for Study 2 groups. Error bars indicate 95% bootstrapped confidence intervals. PTSD = posttraumatic stress disorder; ED = eating disorder; BPD = borderline personality disorder.

There was a large, significant difference in the absolutist index between the Study 1 groups, as determined by a multilevel mixed-effects model, *F*(3, 29) = 71.549, *p* < .001. Using paired comparisons in the mixed-effects model, we compared the control group with each of the Study 1 test groups to assess our first hypothesis. We also compared the suicidal ideation forum group with the remaining two test groups (anxiety and depression forums) to assess our second hypothesis. The mean absolutist index for the control forum group (*M* = 0.97%, *SD* = 0.85) was significantly lower than anxiety (*M* = 1.45%, *SD* = 0.97, *p* < .001, *d* = 3.24, 95% CI = [0.07, 0.11]), depression (*M* = 1.45%, *SD* = 1.0, *p* < .001, *d* = 3.14, 95% CI = [0.08, 0.11]), and suicidal ideation (*M* = 1.80%, *SD* = 1.04, *p* < .001, *d* = 4.56, 95% CI = [0.14, 0.18]) test forum groups. Moreover, the suicidal ideation group was significantly greater than both the anxiety (*p* < .001, *d* = 1.74, 95% CI = [−0.09, −0.05]) and depression (*p* < .001, *d* = 1.71, 95% CI = [−0.09, −0.05]) groups (Fig. 1a). These results are consistent with both of our Study 1 hypotheses. Post hoc comparisons with a Bonferroni correction revealed that there was no significant difference between anxiety and depression forum group means (*p* = 1.00).

#### Multilevel mixed-effects model for the comparison dictionaries

Using the LIWC software, we produced indices for our nonabsolutist dictionary and all 73 LIWC dictionaries. We were interested in determining which comparison dictionary index would produce comparable significance levels and effect sizes to that of our absolutist index. We again conducted a multilevel mixed-effects model and pairwise comparisons for each of the 74 comparison dictionary indices. Table 2 displays the test statistics and effect sizes for the 16 dictionaries with the largest effects. Notably, our absolutist index has satisfied the study hypotheses better than any of the linguistic dimensions previously linked to affective disorder (negative emotions, personal pronouns etc.). Although “negative emotion” words were predictably more prevalent in test group forums than control forums, they paradoxically were less prevalent in suicidal ideation forums than anxiety or depression forums. This was also the case for other content dictionaries like “sad,” affect,” and “feel.”

**Table 2.** Results for Study 1 Paired Comparisons

	*H*_1_						*H*_2_			
	Control < anxiety		Control < depression		Control < suicidal ideation		Anxiety < suicidal ideation		Depression < suicidal ideation	
Dictionary	*d*	*t*	*d*	*t*	*d*	*t*	*d*	*t*	*d*	*t*
Absolutist	3.24	8.57**	3.14	8.48**	4.56	12.43**	1.74	4.62**	1.71	4.60**
Death	1.95	5.02**	2.42	6.29**	8.08	21.37**	5.70	14.82**	5.28	13.82**
Anxiety	10.04	27.21**	2.68	7.37**	0.52	1.44	6.67	−18.27**	1.47	−4.06**
Neg. emo	5.81	15.85**	4.36	11.98**	3.56	9.92**	1.05	−2.90*	0.05	−0.14
Sad	2.02	5.56**	5.18	14.38**	3.70	10.44**	1.78	4.96**	0.51	−1.43
Affect	4.47	12.18**	3.69	10.15**	3.23	9.03**	0.37	−1.02	0.15	0.41
Anger	2.43	6.65**	2.38	6.59**	3.54	9.94**	1.36	3.76*	1.35	3.77*
Certain	1.84	4.89**	2.02	5.43**	3.21	8.78**	1.51	4.07**	1.34	3.63*
Pronouns	2.53	6.96**	2.56	7.10**	2.90	8.12**	0.69	1.92	0.65	1.81
Insight	3.04	8.08**	2.69	7.24**	1.22	3.35*	1.08	−2.92*	0.87	−2.35*
Article	2.41	−6.57**	2.34	−6.43**	2.64	−7.35**	0.57	−1.57	0.60	−1.65
Swear	1.02	2.75*	0.98	2.67*	2.55	7.06**	1.49	4.08**	1.50	4.12**
Feel	2.32	6.36**	2.08	5.72**	1.17	3.27*	0.64	−1.78	0.48	−1.33
Function	1.75	4.83**	2.15	5.97**	2.01	5.63**	0.48	1.33	0.18	0.50
I	1.87	5.15**	1.95	5.37**	1.88	5.22**	0.27	0.74	0.21	0.57
Negate	0.77	2.13*	1.89	5.26**	1.95	5.49**	1.13	3.16*	0.32	0.9

Note: Displayed are 16 dictionaries with the largest effects. For each dictionary, three *t* tests compared the transformed data for the control group index (dictionary % prevalence) to each of the test groups (anxiety, depression, and suicidal ideation forums) to address Hypothesis 1 (*H*_1_). Two *t* tests also compared the suicidal ideation forum group with the remaining two test groups (anxiety and depression) to address Hypothesis 2 (*H*_2_). LIWC dictionaries are ordered according to average Cohen’s *d* effect size. Neg. emo = negative emotions; I = first-person singular pronouns (e.g., *I, me, my*).

* *p* < .05. **p < .001.

#### Analysis of covariance

We ran an analysis of covariance (ANCOVA) to measure the unique predictive validity of absolutist words after partialling out the effects of the negative emotion words, pronouns, and certainty words. Negative emotions and pronouns have previously been identified as strong linguistic markers of affective disorder, and the certainty words index is the most conceptually related to our absolutist index. We found that there was still a significant main effect for the absolutist index between groups, after controlling for the certainty index, negative emotions index, and the pronoun’s index, *F*(3, 3860) = 20.575, *p* < .001. Paired comparisons reveal that all contrasts remained significant to *p* < .01.

#### Confirmatory factor analysis

For Study 1 forums, we calculated indices for each individual *word* in the absolutist and nonabsolutist dictionaries using an in-house python script (full python code is available in the Supplemental Material) and the Natural Language Tool Kit (Bird, Klein, & Loper, 2009). This means that we had the percentage prevalence of each *word* rather than each dictionary. Using these data, we conducted a confirmatory factor analysis on the combined list of 65 absolutist and nonabsolutist words with a direct oblimin rotation and a loadings cutoff > 0.55. We found that the highest loading words on the first factor were all absolutist except for *really* (which is an adverbial intensifier) and *anything*, which we had originally categorized as absolutist but, because of a lack of independent expert validation, was moved to the nonabsolutist dictionary. The highest loading words on Factor 2 were all nonabsolutist except for the absolutist word *definitely*. Other than *definitely*, no absolutist word loaded outside of Factor 1. The factor analysis was not able to separate “extreme words” from nonabsolutist words (see Table S3 in the Supplemental Material). To examine the absolutism factor further, we used structural equation modeling to test the model fit of the seven highest loading words on Factor 1 from the factor analysis. Model fit was assessed using AMOS version 24 (SPSS). A seven-item, one-factor model adequately fit the data (χ^2^ = 14.461, *df* = 14, goodness of fit index = .912, comparative fit index = .996, normed fit index = .903). Including more words in the model reduced the model fit below generally accepted levels.

#### Sensitivity analysis

The smallest group in this study is the suicidal ideation group. Inferences about this group are based on data from 368 members in four separate suicidal ideation forums. Moreover, these forums may be perceived as less conventional than others used in this research. For this reason, we conducted a sensitivity analysis to ensure the results obtained from this group are robust. The multilevel mixed-effects model for the absolutist index was recalculated after sequentially excluding all data from each of the suicidal ideation forums in turn. This produced four sets of test statistics, each with one suicidal ideation forum excluded. Paired comparisons showed that the absolutist index for the suicidal ideation group remained significantly greater than the control group (*p*s < .001, *d*s = 3.85–4.41), the anxiety group (*p*s < .001, *d*s = 1.39–1.71), and the depression group (*p*s < .001, *d*s = 1.37–1.69). The narrow range of effect sizes for each comparison confirms that these findings are robust, and not driven by a forum outlier in the suicidal ideation group.

### Study 2

#### Multilevel mixed-effects model for the absolutist index

Our third hypothesis predicted that mental health forum groups strongly associated with absolutist thinking (BPD and ED) would use more absolutist words than mental health forum groups less associated with absolutist thinking (PTSD and schizophrenia). A multilevel mixed-effects analysis found that there was a significant difference in the absolutist index between Study 2 groups, *F*(3, 16) = 5.515, *p* = .009. Paired comparisons revealed that the mean absolutist index for the BPD forum group (*M* = 1.47, *SD* = 1.01) was significantly greater than the PTSD (*M* = 1.13, *SD* = 0.83, *p* < .001, *d* = 0.36, 95% CI = [−0.07, −0.02]) and the schizophrenia forum groups (*M* = 1.14, *SD* = 0.86, *p* < .001, *d* = 0.35, 95% CI = [−0.07, −0.03]). They also revealed that the absolutist index of the ED forum group (*M* = 1.25, *SD* = 0.95) was significantly greater than the schizophrenia (*p* = .009, *d* = 0.12, 95% CI = [−0.04, −0.001]) but not PTSD (*p* = .081, *d* = 0.13, 95% CI = [−0.03, 0.01]) forum groups (Fig. 1b). A critical assumption in this contrast, is that the control and test groups have similar levels of psychological distress. We sought to verify this assumption using the LIWC negative emotions dictionary. A paired comparison found no significant difference in the mean negative emotions index between the Study 2 control (*M* = 3.51, *SD* = 2.02) and test (*M* = 3.71, *SD* = 1.76, *p* = .335) forum groups (Fig. 1c). Therefore, it seems that absolutism is associated with certain types of psychopathology forums and not psychological distress forums *per se*.

#### Comparison of Study 1 with Study 2

In comparing the absolutist index of Study 1 and 2 groups, post hoc comparisons with a Bonferroni correction revealed that the suicidal ideation forum group had an index significantly greater than ED and BPD forum groups (*p* < .001). ED but not BPD had an index significantly lower than anxiety and depression forum groups (*p*s = .001). Study 2 control forum groups PTSD and schizophrenia had an index significantly lower than all Study 1 test forum groups (*p*s < .001).

#### Sensitivity analysis

The smallest group in this study is the BPD group. Inferences about this group are based on data from 326 members in four separate BPD forums. This group also produced the most extreme absolutist index scores. Once again, we conducted a sensitivity analysis to ensure the results obtained from this group are robust. The multilevel mixed-effects model for the absolutist index was recalculated after sequentially excluding all data from each of the BPD forums in turn. This produced four sets of test statistics, each with one BPD forum excluded. Paired comparisons show that the absolutist index for the BPD group remained significantly greater than the PTSD group (*p*s < .026, *d*s = 1.25–1.91) and the schizophrenia group (*p*s < .008, *d*s = 1.56–2.24). Once again, the positive findings from the smallest group in the study appear to be robust and not dependent on any single forum outlier.

### Study 3

#### Multilevel mixed-effects model for the absolutist index

**Fig. 2.**
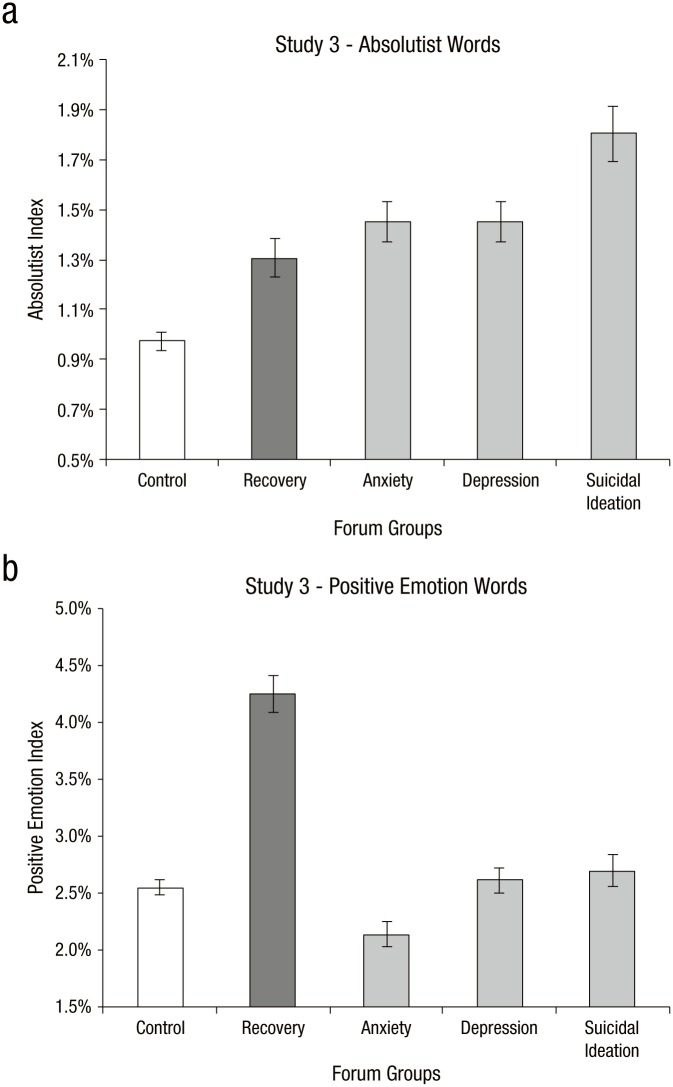
Mean percentage of (a) absolutist words and (b) positive emotion words for the recovery group and all Study 1 groups (control, anxiety, depression, suicidal ideation). Error bars indicate 95% bootstrapped confidence intervals.

Our final hypothesis predicted that the recovery forum group would use significantly more absolutist words than the Study 1 control forum group. Paired comparisons in a multilevel mixed-effects model found that the mean absolutist index of the recovery forum group (*M* = 1.31%, *SD* = 0.95) was significantly greater than the Study 1 control forum group (*p* < .001, 95% CI = [−0.09, −0.05], *d* = 0.37). Paired comparisons also found a significant difference in the absolutist index between the recovery forum group and the anxiety group (*p* = .018, 95% CI = [−0.001, 0.04], *d* = 0.15) and depression group (*p* = .018, 95% CI = [−0.001, 0.04], *d* = 0.15). Like the anxiety and depression groups, the recovery group also had a significantly lower absolutist index than the suicidal ideation group (*p* < .001, 95% CI = [−0.06, −0.12], *d* = 0.50). Although the absolutist index of the recovery group was significantly different from anxiety and depression groups, the more accurate bias-corrected CIs reveal that the differences are marginal; relative effect sizes reveal that the recovery group absolutist index is closer to anxiety and depression (*d* = 0.15) than to the control group (*d* = 0.37; Fig. 2a). We noted earlier that the contents of the recovery forums were very positive. To illustrate this fact, we ran the LIWC positive emotions dictionary on the above groups (Fig. 2b). There was indeed a very large difference in the prevalence of positive emotions. Paired comparisons found that the recovery forum group contained more positive emotion words than all the remaining groups (*p*s < .001).

#### Sensitivity analysis

Although the recovery group is relatively large, with 558 members in 7 different forums, this group is somewhat unconventional and the number of members in each forum were somewhat unequal (see Table S1). We therefore deemed it appropriate to conduct another sensitivity analysis to ensure the results obtained from this group are robust. The multilevel mixed-effects model for the absolutist index was recalculated after sequentially excluding all data from each of the recovery forums in turn. This produced seven sets of test statistics, each with one recovery forum excluded. Paired comparisons show that the absolutist index for the recovery group remained significantly greater than the control group (*p*s < .001, *d*s = 1.88–2.30). This again confirms that the positive findings from this group are robust and not dependent on any single forum outlier.

## Discussion

### Main findings

The data we have presented confirm that the use of absolutist words is elevated in the natural language of various affective disorder forum groups. As expected, in Study 1 we found that the percentage of absolutist words in anxiety, depression, and suicidal ideation test groups was significantly greater than in control groups (*H*_1_), and that the percentage of absolutist words in the suicidal ideation forum group was significantly greater than in both the anxiety and depression forum groups (*H*_2_). These findings have support from a previous study, Fekete (2002) used an adapted Weintraub text analysis method on four Internet forums (suicide, depression, anxiety, and a journalism control). They found significant results for 13 language variables including negations and dichotomous expressions. Our first study has built on this preliminary finding, using a wider range of more rigorous controls, a larger corpus of data, and a hypothesis-driven study design.

In Study 2, consistent with our expectations, we found the absolutist index was greater for BPD and ED forums than PTSD and schizophrenia forums, although this did not reach significance between ED and PTSD. All four mental health groups contained similar amounts of negative emotion terms, but only BPD and ED are strongly associated with absolutist thinking. This suggests that our index is more sensitive to absolutism than psychological distress.

In Study 3, we proposed that if the absolutist index for the recovery forums was similar to depression forums, this would suggest that absolutist thinking has some trait-like qualities that persist outside of depressive episodes. This is indeed what we observed. Even though the recovery forums were largely very positive, the percentage of absolutist words in the recovery group had overlapping CIs with both the anxiety and depression forum groups, and was significantly greater than the control forum group. It is widely acknowledged that an episode of depression increases the risk of future depressive episodes (Teasdale et al., 2000). In many ways, preventing this recurrence is the focus of most treatments. Consequently, there is keen interest in identifying potential cognitive vulnerability factors which are observed during episodes of depression and persist even after the episode has ended. Our findings indicate that absolutism may be such a vulnerability factor. The “scar hypothesis” (Lewinsohn, Steinmetz, Larson, & Franklin, 1981) provides a different explanation. Here the depressive episode itself alters the linguistic style/vocabulary of the individual, this then persists as a “scar” after the depressive episode has abated.

### Comparison with other dictionaries

Text analysis research on written data from depressed and suicidal individuals has repeatedly shown elevated use of negative emotion words and pronouns (for review, see Tausczik & Pennebaker, 2010). We also found these to be strong markers of affective disorder in the present study. However, we have paradoxically found that “negative emotions,” “sad,” “affect,” and “feel” dictionaries were more prevalent in anxiety and depression than the suicidal ideation group. This is inconsistent with the belief that suicidal individuals have a greater amount of negative emotions (de Klerk et al., 2011; Orbach, Mikulincer, Gilboa-Schechtman, & Sirota, 2003; Stein, Apter, Ratzoni, Har-Even, & Avidan, 1998), and some research has previously shown that “negative emotion [words] use tends to increase approaching suicide” (Pennebaker & Chung, 2013). These mixed findings only reaffirm that “function” words are a better gauge of thinking processes than “content” words (Chung & Pennebaker, 2007). Our absolutist dictionary also produced larger effects than pronouns (and its first-person singular subcategory), which had previously been identified as better markers of affective disorder than negative emotion words (Pennebaker & Chung, 2013).

The LIWC “certainty” index (Table 2) is the most closely related to our absolutist index, comprising words that denote high or total certainty. Although indeed similar, the certainty index does not include some words that are absolutist (i.e., “nothing”) and contains others that are not (i.e., “frankly”). Moreover, unlike our absolutist dictionary, many of its component words are not neutrally valenced (i.e., perfect).

Finally, we found that “swear” words produced a similar significance pattern to absolutist words (Table 2). Swear words are commonly used as adverbial intensifiers (Peters, 1994; Romero, 2013). For example, instead of writing “I’m *completely* sick of this,” depressed/suicidal individuals may write something akin to “I’m *fucking* sick of this,” replacing the absolutist word “completely” with something even more forceful, both functionally serving as adverbial intensifiers of the strongest kind.

### Absolute versus extreme

Previous studies have often used the terms absolute and extreme interchangeably (e.g., Teasdale et al., 2001). A central assumption in the present research is that absolutist words are uncorrelated with extreme words; this assumption was tested. We found that only 25% of absolutist words were also deemed extreme by some of the independent expert judges. Moreover, none of the words we had categorized as extreme were deemed absolutist, with the single exception of *really*, which was categorized as absolutist by one out of the five judges. This was reaffirmed by the confirmatory factor analysis (Table S3), in which only words we had categorized as absolutist loaded onto Factor 1, with the single exception, once again, of the adverbial intensifier *really*. We believe that a clear distinction should be made between these two concepts in future research; and that the terms should not be used interchangeably.

### Anxiety and depression within control groups

Individuals with cancer, PTSD, and schizophrenia have high levels of comorbid anxiety and depression. This might lead us to expect a higher absolutist index for these forum groups. However, the cancer group produced an absolutist index identical to the other Study 1 control groups; and the PTSD and schizophrenia groups had a significantly lower absolutist index than all Study 1 test groups. This may be because symptoms of anxiety and depression in cancer, PTSD, and schizophrenia have a known specific cause, namely, having cancer, PTSD, or schizophrenia. One does not have to be absolutist, or even disposed to affective disorder, to experience feelings of anxiety or depression about a brain tumor, a traumatic event, or hallucinations. In contrast, anxiety and depression disorders often have multiple vague or even unknown causes. Predisposed individuals are pushed into anxiety and depression by circumstances that by necessity would not have the same effect in the general population.

### Implications

The maladaptive status of absolutist thinking is a recognized part of cognitive therapy (CT; C. Williams & Garland, 2002). To date, theoretical and anecdotal support has mostly served as the basis for its inclusion; we hope the findings from our studies will add empirical justification. The extent to which absolutist thinking is currently addressed by CT depends on the form of CT used and the preferred methods of each practitioner. For example, combatting absolutist thinking is at the very core of rational-emotive behavioral therapy (David, Lynn, & Ellis, 2009), whereas reducing negative thoughts takes primacy in other forms of CT. Recently, research into treating cognitive vulnerabilities and preventing relapse has migrated toward the new “third-wave” therapies (Teasdale et al., 2000). These therapies, such as mindfulness-based cognitive therapy and acceptance and commitment therapy, are largely geared toward increasing cognitive flexibility (e.g., Kahl, Winter, & Schweiger, 2012). Our findings are therefore in step with the recent trend toward cultivating adaptive cognitive *processes* (i.e., flexibility) as distinct from changing the *content* of thoughts (i.e., negativity).

### Limitations and future directions

Because this study had large samples from multiple sources, and a naturalistic observational design, it consequently had low experimental control. For example, we could only infer general demographic characteristics from different forums (e.g., women post on Mumsnet and young people post on Student Room). Usernames served to distinguish members, however it is possible that some members might post using more than one profile or use different usernames for different forums. Fundamentally, the identities and motivation of users are largely unknowable, and this is an inevitable limitation in this study. As outlined in the methods, we did check that the authors of posts were at least purporting to be a representative of the relevant online community, but we had no power to go beyond this basic check. Follow-up studies could use an experimental study design, and perhaps alternative methodologies, to replicate and extend the findings initially presented here. Despite likely being limited to a smaller sample size and perhaps lacking ecological validity, such studies would be able to control participant characteristics, writing topics and the setting.

Our findings in this study relate to differences between groups, such an analysis provides important insights into the markers associated with affective disorder. However, in this research, we have not addressed within-person variation in absolutist thinking and how that relates to changes in affective symptoms at an individual level (cf. Molenaar & Campbell, 2009). For example, are individual changes in suicidal ideation over time reflected in changes in use of absolutist words? Future research could seek to track absolutist thinking (and affective disorder) in individuals over time. This could have even greater utility for clinical practice.

In measuring aggregate differences in absolutist words between groups we have not examined the specific nature of the relationship. Although we present data that may point to absolutism as a possible cognitive vulnerability factor, the extent and mechanism of any causal role are not addressed here. Future intervention studies could examine the causal status of absolutist thinking; one possibility would be to use a cognitive bias modification paradigm (Hallion & Ruscio, 2011). The aim would be to introduce some manipulation of absolutist thinking in participants and then examine the subsequent effects. Alternatively, a narrow form of cognitive behavioral therapy that focuses on targeting absolutist thinking could be clinically trialed.

## Supplemental Material

Al-MosaiwiMultilevel_linear_mixed_effects_model_syntax_Supplemental_Material – Supplemental material for In an Absolute State: Elevated Use of Absolutist Words Is a Marker Specific to Anxiety, Depression, and Suicidal Ideation Click here for additional data file. Supplemental material, Al-MosaiwiMultilevel_linear_mixed_effects_model_syntax_Supplemental_Material for In an Absolute State: Elevated Use of Absolutist Words Is a Marker Specific to Anxiety, Depression, and Suicidal Ideation by Mohammed Al-Mosaiwi and Tom Johnstone in Clinical Psychological Science

## Supplemental Material

Al-Mosaiwi_Open_Practices_Disclosure – Supplemental material for In an Absolute State: Elevated Use of Absolutist Words Is a Marker Specific to Anxiety, Depression, and Suicidal Ideation Click here for additional data file. Supplemental material, Al-Mosaiwi_Open_Practices_Disclosure for In an Absolute State: Elevated Use of Absolutist Words Is a Marker Specific to Anxiety, Depression, and Suicidal Ideation by Mohammed Al-Mosaiwi and Tom Johnstone in Clinical Psychological Science

## Supplemental Material

Code_Supplemental_Material – Supplemental material for In an Absolute State: Elevated Use of Absolutist Words Is a Marker Specific to Anxiety, Depression, and Suicidal Ideation Click here for additional data file. Supplemental material, Code_Supplemental_Material for In an Absolute State: Elevated Use of Absolutist Words Is a Marker Specific to Anxiety, Depression, and Suicidal Ideation by Mohammed Al-Mosaiwi and Tom Johnstone in Clinical Psychological Science

## Supplemental Material

Table_S1_Supplemental_Material – Supplemental material for In an Absolute State: Elevated Use of Absolutist Words Is a Marker Specific to Anxiety, Depression, and Suicidal Ideation Click here for additional data file. Supplemental material, Table_S1_Supplemental_Material for In an Absolute State: Elevated Use of Absolutist Words Is a Marker Specific to Anxiety, Depression, and Suicidal Ideation by Mohammed Al-Mosaiwi and Tom Johnstone in Clinical Psychological Science

## Supplemental Material

Table_S2_Supplemental_Material – Supplemental material for In an Absolute State: Elevated Use of Absolutist Words Is a Marker Specific to Anxiety, Depression, and Suicidal Ideation Click here for additional data file. Supplemental material, Table_S2_Supplemental_Material for In an Absolute State: Elevated Use of Absolutist Words Is a Marker Specific to Anxiety, Depression, and Suicidal Ideation by Mohammed Al-Mosaiwi and Tom Johnstone in Clinical Psychological Science

## Supplemental Material

Table_S3_Supplemental_Material – Supplemental material for In an Absolute State: Elevated Use of Absolutist Words Is a Marker Specific to Anxiety, Depression, and Suicidal Ideation Click here for additional data file. Supplemental material, Table_S3_Supplemental_Material for In an Absolute State: Elevated Use of Absolutist Words Is a Marker Specific to Anxiety, Depression, and Suicidal Ideation by Mohammed Al-Mosaiwi and Tom Johnstone in Clinical Psychological Science
