# Supplementary material for: In an Absolute State: Elevated Use of Absolutist Words Is a Marker Specific to Anxiety, Depression, and Suicidal Ideation
Source: Clin Psychol Sci. 2018 Jan 5;6(4):529–42. doi: 10.1177/2167702617747074 (PMC6376956; doi:10.1177/2167702617747074)
Supplement: Code_Supplemental_Material – Supplemental material for In an Absolute State: Elevated Use of Absolutist Words Is a Marker Specific to Anxiety, Depression, and Suicidal Ideation [file Code_Supplemental_Material.pdf]

In-House Text Analysis Python Code

In house Python Script for Word Level Analysis:

```
# script to assess absolutism/reletivism

from __future__ import division
import nltk
import nltk, re, pprint
import numpy
import matplotlib
import re
import xlsxwriter
import string
import codecs
from nltk.stem.porter import PorterStemmer
porter_stemmer = PorterStemmer()

workbook = xlsxwriter.Workbook('absolutist001.xlsx') #creating a workbook
worksheet = workbook.add_worksheet() #creating a worksheet

number_list = []

file = open('absol19.txt') # This is the dictionary in use - change as
appropriate
t = file.read(); #reading file
t.lower() # Normalising
u_t = unicode(t, errors='ignore') #converting string into Unicode
absol = nltk.word_tokenize(u_t) #tokenize the 'string' -> absol
text = nltk.Text(absol) #converts to nltk?
sorted(set(absol)) #sorts into alphabetical order and removes repeats

file = open('suic.txt') #This is the file you want to test
raw = file.read(); #Reading above text
u_raw = unicode(raw, errors='ignore') #Converting to Unicode
u_raw.lower() #Normalising
Ellis = nltk.word_tokenize(u_raw) #Tokenising text
text = nltk.Text(Ellis) #converts to nltk

for item in absol: #for each item in absol list
    fdist = nltk.FreqDist(Ellis) #calculates frequency distribution for all words in
Ellis
    numbers = str(fdist.freq(item)) #Convert dictionary item frequencies to
string format
    re.split(r'\s+', numbers) # convert frequency string to list
    number_list.append(numbers) #add those numbers to a numbers list

row = 0
col = 0
```

```
for item in number_list:
    worksheet.write(row, col,     item)
    row += 1

worksheet.write(row, 1, 'Total')
worksheet.write(row, 0, '=SUM(A1:A431)')

workbook.close()
#below code prints out concordance
def get_all_phrases_containing_tar_wrd(target_word, tar_passage,
left_margin = 10, right_margin = 10):
    Ellis = nltk.word_tokenize(tar_passage)
    text = nltk.Text(Ellis)
    c = nltk.ConcordanceIndex(text.Ellis, key = lambda s: s.lower())
    concordance_txt = ([text.Ellis[map(lambda x: x-5 if (x-left_margin)&gt[0]
else 0, [offset])[0]:offset+right_margin]
                        for offset in c.offsets(target_word)])
    return [''.join([x+' ' for x in con_sub]) for con_sub in concordance_txt]

Ellis = nltk.word_tokenize(u_raw)# This section prints out only matched words
ci = nltk.ConcordanceIndex(Ellis)
for t_word in absol:
    if ci.offsets(t_word):
        ci.print_concordance(t_word)
print
print 'Results from function'
results = get_all_phrases_containing_tar_wrd(absol, u_raw)
for result in results:
   print result
```
