# Supplementary material for: In an Absolute State: Elevated Use of Absolutist Words Is a Marker Specific to Anxiety, Depression, and Suicidal Ideation
Source: Clin Psychol Sci. 2018 Jan 5;6(4):529–42. doi: 10.1177/2167702617747074 (PMC6376956; doi:10.1177/2167702617747074)
Supplement: Table_S1_Supplemental_Material – Supplemental material for In an Absolute State: Elevated Use of Absolutist Words Is a Marker Specific to Anxiety, Depression, and Suicidal Ideation [file Table_S1_Supplemental_Material.pdf]

Table S1. Characteristics of Test and Control Internet Forums.

Study	Group	Category	Forum source	Word Count[b]	No. of posts[c]	Avg. post length[d]
Study 1	Control Groups	**General Forums[a]**	Mumsnet.com	36617	157	210
			Boards.ie/TheLadiesLounge	31420	131	221
			Boards.ie/TheGentlemensClub	26419	121	173
			Askmen.com	37222	141	258
			PensionersForum.co.uk	30102	126	154
			TheStudentRoom.co.uk	31175	142	221
			Boards.ie/WorkandJobs	32254	109	258
		**Asthma Forums**	HealthUnlocked.com	33675	122	272
			Patient.info/Asthma	19010	95	211
			Dailystrength.org	34627	90	253
			Healthboards.com	32500	111	248
		**Diabetes Forums**	Diabetes.co.uk	34963	152	211
			Patient.info/Diabetes	32885	139	267
			Diabetessupport.co.uk	38347	174	198
			Diabetes-support.org.uk	31453	122	242
		**Cancer Forums**	Beatingbowelcancer.org	35940	95	259
			Macmillan.org.uk	33042	141	203
			Cancerforums.net	33007	122	225
			ProstateCancerUK.org	33438	93	241
	Test Groups	**General Anxiety Disorder Forums**	Patient.info/Anxiety	42078	152	240
			Anxietyforum.net	38962	85	371
			Anxietyzone.com	43817	115	332
			Nomorepanic.co.uk	32723	90	266
			Mentalhealthforum.net	36387	96	334
			Psychforums.com	35432	76	449
		**Depression Forums**	Patient.info/Depression	34616	103	304
			Mentalhealthforum.net	33167	69	357
			Depressionforums.org	36504	83	341
			Dealingwithdepression.co.uk	30465	65	317
			Psychcentral.com/Depression	40147	107	309
			Beyondblue.org.au	35586	127	256
		**Suicidal Ideation Forums**	Suicideforum.com	42339	112	359
			Takethislife.com	43611	98	383
			Suicidemethods.net	39447	104	355
			Experienceproject.com	38542	59	290
Study 2	Control Group	**Post Traumatic Stress Disorder Forums**	Myptsd.com	40511	112	362
			Psychforums.com/PTSD	38577	90	433
			Psychcentral.com/PTSD	34852	88	387
			Patient.info/PTSD	20659	42	492
			Mentalhealthforum.net	40435	96	400
			Ehealthforum.com	34731	107	302
		**Schizophrenia Forums**	Psychforums.com	38924	77	512
			Schizophrenia.com	33460	106	216
			Mentalhealthforum.net	32687	104	314
			Psychcentral.com	40187	137	277
			eHealthforum.com	36745	75	477
			Healthboards.com	31430	94	314
	Test Group	**Borderline Personality Disorder Forums**	Psychforums.com	35472	98	362
			Mentalhealthforum.net	33589	86	377
			Psychcentral.com	32717	100	312
			Experienceproject.com	15058	42	350
		**Eating Disorder Forums**	Patient.info/Eating-disorders	37018	134	270
			Nationaleatingdisorders.org	35733	126	267
			Mentalhealthforum.net	33433	117	283
			Psychcentral.com	12379	44	281
			Recoveryourlife.com	34152	126	269
Study 3	Test Group	**Recovery Forums**	Depressionforums.org	45054	112	302
			Psychcentral.com	13612	25	378
			Dealingwithdepression.co.uk	12325	24	342
			Takethislife.com/Success Stories	35061	68	455
			Takethislife.com/GettingBetter	36792	94	287
			Beyondblue.org.au/StayingWell	42731	104	375
			Suicideforum.com/PositiveFeelings	48436	131	327

Note. GAD = Generalized anxiety disorder; PTSD = Post traumatic stress disorder; BPD = Borderline personality disorder; ED = Eating disorder.

[a] General Forums = 'Mumsnet' (Women), 'The Ladies Lounge' (Women), 'The Gentlemen's Club' (Men), 'Ask Men' (Men), 'Pensioners Forum' (Elderly), 'Student Room' (Young), 'Work Problems'.

[b] Word count for each forum, only 'first posts' collected.

[c] Number of 'first posts' which comprise the forums corpus.

[d] Average number of words in each forum post
