# Supplementary material for: In an Absolute State: Elevated Use of Absolutist Words Is a Marker Specific to Anxiety, Depression, and Suicidal Ideation
Source: Clin Psychol Sci. 2018 Jan 5;6(4):529–42. doi: 10.1177/2167702617747074 (PMC6376956; doi:10.1177/2167702617747074)
Supplement: Table_S2_Supplemental_Material – Supplemental material for In an Absolute State: Elevated Use of Absolutist Words Is a Marker Specific to Anxiety, Depression, and Suicidal Ideation [file Table_S2_Supplemental_Material.pdf]

Table S2. List of 19 Independently Validated Absolutist Words

Absolutist Words	
**1**	absolutely
**2**	all
**3**	always
**4**	complete
**5**	completely
**6**	constant
**7**	constantly
**8**	definitely
**9**	entire
**10**	ever
**11**	every
**12**	everyone
**13**	everything
**14**	full
**15**	must
**16**	never
**17**	nothing
**18**	totally
**19**	whole
