# Supplementary material for: In an Absolute State: Elevated Use of Absolutist Words Is a Marker Specific to Anxiety, Depression, and Suicidal Ideation
Source: Clin Psychol Sci. 2018 Jan 5;6(4):529–42. doi: 10.1177/2167702617747074 (PMC6376956; doi:10.1177/2167702617747074)
Supplement: Table_S3_Supplemental_Material – Supplemental material for In an Absolute State: Elevated Use of Absolutist Words Is a Marker Specific to Anxiety, Depression, and Suicidal Ideation [file Table_S3_Supplemental_Material.pdf]

Table S3. Highest Loading Words in the Confirmatory Factor Analysis.

	Components	
FACTORS	**1**	**2-5**
everything	0.864	
ever	0.725	
always	0.717	
nothing	0.684	
anything[a]	0.68	
never	0.634	
really[b]	0.602	
completely	0.594	
every	0.559	
about		0.677
huge		0.636
generally		0.625
often		0.611
some		0.602
somewhat		0.589
slight		0.576
might		0.576
definitely[c]		0.573
nearly		0.562

Note. Individual absolutist and non-absolutist words factored according to their study 1 forums indices. [a] Italicized word not categorized as absolutist by independent expert judges. [b] Italicized word not categorized as absolutist by independent expert judges. [c] Italicized word categorized as absolutist by independent expert judges.
